# Real-Time Small UAV Detection in Complex Airspace Using YOLOv11 with Residual Attention and High-Resolution Feature Enhancement

**DOI:** 10.3390/jimaging12030140

**Published:** 2026-03-20

**Authors:** Chuang Han, Md Redwan Ullah, Amrul Kayes, Khalid Hasan, Md Abdur Rouf, Md Rakib Hasan, Shen Tao, Guo Gengli, Mohammad Masum Billah

**Affiliations:** 1 Heilongjiang Province Key Laboratory of Laser Spectroscopy Technology and Application, Harbin University of Science and Technology, Harbin 150080, China; hanchuang@hrbust.edu.cn (C.H.); iamredwanullah@gmail.com (M.R.U.); 2322200005@stu.hrbust.edu.cn (K.H.); 2 Sunny Group Co., Ltd., Yuyao 315400, China; jtwanghf@sunnyoptical.com; 3 School of Computing and Artificial Intelligence, Southwest Jiaotong University, Chengdu 610031, China; iamamrulkayes@gmail.com; 4 School of Electronics and Information Engineering, Hebei University of Technology, Tianjin 300130, China; abdurrouf.cse@gmail.com; 5 Department of Marketing, Begum Rokeya University, Rangpur 5400, Bangladesh; engr.mdrakibhasan@gmail.com; 6 Higher Educational Key Laboratory for Measuring, Control Technology, and Instrumentation of Heilongjiang Province, Harbin 150080, China; masumbillah42.75@gmail.com

**Keywords:** drone detection, Unmanned Aerial Vehicle (UAV), YOLOv11, real-time object detection, residual attention module

## Abstract

Detecting small unmanned aerial vehicles (UAVs) in complex airspace presents significant challenges due to their minimal pixel footprint, resemblance to birds, and frequent occlusion. To address these issues, we propose YOLOv11-ResCBAM, a novel real-time detection framework that integrates a Residual Convolutional Block Attention Module (ResCBAM) and a high-resolution P2 detection head into the YOLOv11 architecture. ResCBAM enhances channel and spatial feature refinement while preserving original feature contexts through residual connections, and the P2 head maintains fine spatial details crucial for small-object localization. Evaluated on a custom dataset of 4917 images (11,733 after augmentation) across three classes (drone, bird, airplane), our model achieves a mean average precision at the 0.5–0.95 IoU threshold (mAP@0.5–0.95) of 0.845, representing a 7.9% improvement over the baseline YOLOv11n, while maintaining real-time inference at 50.51 FPS. Cross-dataset validation on VisDrone2019-DET and UAVDT benchmarks demonstrates promising generalization trends. This work demonstrates the effectiveness of the proposed approach for UAV surveillance systems, balancing detection accuracy with computational efficiency for deployment in security-critical environments.

## 1. Introduction

In recent years, small unmanned aerial vehicles (UAVs), commonly known as drones, have rapidly emerged as versatile tools across a wide range of domains, including logistics, precision agriculture, infrastructure inspection, environmental monitoring, military reconnaissance, and public safety [1,2]. Their relatively low cost, compact size, and ease of operation have driven their mass adoption by both civilian and commercial users. However, the widespread availability of UAVs has also raised critical concerns regarding their potential misuse [3]. Incidents of unauthorized aerial surveillance, cross-border smuggling, and intrusion into restricted airspace have become increasingly frequent. As a result, there is a growing demand for robust and real-time UAV detection systems capable of identifying and tracking small drones before they pose a threat [4].

Accurate and early detection of small UAVs is essential for securing urban environments, safeguarding critical infrastructure, and managing congested airspace [5]. Despite these advancements, detecting small UAVs in real-world conditions is inherently challenging. Unlike larger aircraft, small drones typically exhibit minimal radar cross-sections, making them difficult to identify using conventional radar systems [6,7]. Visually, they often resemble birds or commercial aircraft, further complicating detection through optical or infrared sensors. Additional obstacles such as occlusion, variable lighting, adverse weather conditions, and background clutter reduce the reliability of traditional vision-based detection systems [8,9]. Even state-of-the-art convolutional neural network (CNN) models struggle when small objects occupy limited pixel space or appear in complex backgrounds. Several deep learning-based object detectors have been developed to address general detection tasks, with YOLO (You Only Look Once) emerging as one of the most widely adopted frameworks due to its high-speed, single-shot inference capability [10,11].

Despite recent improvements in YOLO-based detectors, two key limitations remain for UAV detection in complex airspace: (1) loss of fine spatial features caused by progressive downsampling, and (2) the lack of attention mechanisms that preserve original feature representations while refining discriminative information. Existing approaches typically apply attention sequentially, which may suppress useful contextual information and degrade detection stability for small targets. To address these challenges, we propose YOLOv11n-ResCBAM, a detection framework that combines a residual attention mechanism with a high-resolution detection head to improve feature representation and small-object localization. As summarized in Table 1, most existing YOLO-based UAV detection approaches rely on sequential attention mechanisms or dataset-specific optimizations, while few studies explore residual attention structures combined with high-resolution detection heads [12,13,14].

Recent iterations, such as YOLOv5 and YOLOv8, have introduced improved architecture designs and attention mechanisms to enhance performance [15]. Though promising, these models still face difficulties when applied to small-object detection in aerial imagery. The primary limitations include progressive downsampling, which erodes fine spatial detail, and the lack of dedicated high-resolution detection layers optimized for small-scale features. While attention modules like the Convolutional Block Attention Module (CBAM) have shown promise in refining feature maps, their integration into lightweight, real-time architectures remains limited. Significantly, existing public UAV datasets often lack class diversity, occlusion cases, and background variability necessary for real-world generalization [16].

To address these challenges, this research proposes YOLOv11n-ResCBAM, a real-time object detection framework specifically designed to improve the detection of small UAVs in visually complex environments. The model builds upon the YOLOv11 architecture and introduces two key innovations: (1) a high-resolution P2 detection head to preserve fine-grained spatial information often lost during feature downsampling, and (2) a Residual Convolutional Block Attention Module (ResCBAM) that combines channel and spatial attention mechanisms within a residual structure to enhance semantic feature selection without sacrificing the original context [17]. These enhancements aim to bridge the gap between high-speed inference and precise small-object detection. To the best of our knowledge, no prior work combines a residual channel–spatial attention mechanism with a high-resolution P2 detection head within the YOLOv11 framework for real-time UAV detection, particularly under complex airspace conditions.

### The Key Contributions of This Research Are as Follows

- Development of the YOLOv11-ResCBAM architecture, which integrates residual attention modules and a high-resolution detection head into the YOLOv11 backbone to improve UAV detection accuracy and localization.
- Construction of a custom multi-class UAV dataset comprising 4917 images (11,733 after augmentation) across drone, bird, and airplane classes, including a robust test set of 506 images to ensure reliable evaluation under diverse environmental and occlusion conditions.
- Quantitative performance analysis demonstrating that the proposed model achieves precision of 0.968, recall of 0.921, and mAP@0.5 of 0.969 at 50.51 FPS, with mAP50-95 of 0.845, representing a 7.9% improvement over the baseline YOLOv11n. The model demonstrates improved localization precision across IoU thresholds while maintaining real-time capability.
- An effective attention integration strategy, in which ResCBAM modules are selectively applied to YOLOv11 backbone layers containing cv1 and cv2, enabling a balance between computational efficiency and feature refinement.
- Demonstrated application potential for use in smart surveillance systems, autonomous drone traffic control, and real-time airspace security solutions, particularly in scenarios where small-object detection is critical.

This work provides a practical and scalable solution to the increasingly urgent problem of small UAV detection in complex environments, combining architectural innovations with real-world deployment readiness. The remainder of this paper is organized as follows: Section 2 presents a detailed review of the existing literature on small object detection, attention mechanisms, and drone detection models, highlighting key limitations and research gaps. Section 3 describes the methodology, including the YOLOv11n-ResCBAM model architecture, the integration of residual attention modules, the high-resolution P2 detection head, dataset preparation, training procedures, and evaluation metrics. Section 4 outlines the experimental results, including ablation studies, visual diagnostics, and comparisons with state-of-the-art YOLO variants. Section 5 provides a critical discussion of the findings, emphasizing practical implications, trade-offs between accuracy and speed, and deployment considerations. Finally, Section 6 concludes the paper with key contributions and future research directions aimed at enhancing model robustness and deployment efficiency in real-world UAV detection scenarios.

## 2. Related Works

Real-time object detection, particularly for small aerial targets, is a key challenge in UAV-based computer vision applications. One-stage detectors such as YOLO have become widely used due to their speed and efficiency. On the other hand, conventional versions often struggle with detecting small objects in complex aerial scenes due to the loss of fine-grained features and background interference. Several studies have proposed enhancements to YOLO architectures. For instance, an improved YOLOv7 model incorporates non-strided convolution, deformable attention, and efficient multi-scale attention to enhance detection performance for small UAV targets [18,19]. Another research optimized YOLOv8 with CBAM, GAM, and ECA attention modules, resulting in increased precision with limited computational overhead [20]. Temporal modeling has also gained traction. A video-based approach integrates a temporal attention gated recurrent unit (TA-GRU) and deformable transformers into YOLOv7 to leverage spatiotemporal context, improving mAP on the VisDrone2019-VID dataset while maintaining real-time inference [21]. The VisDrone dataset has become a benchmark for evaluating UAV detection models, especially under conditions involving occlusions, dense backgrounds, and small target sizes [12]. Performance in this challenge continues to reveal the limitations of standard architectures in real-world aerial imagery. Transformer-based object detection has emerged as a powerful alternative to CNN-based approaches. Deformable DETR addresses the high computational cost and slow convergence of original DETR by introducing deformable attention modules that focus on relevant spatial locations [22]. DINO-DETR further improves detection performance through contrastive denoising training and mixed query selection [23].

These transformer architectures have shown competitive performance on general object detection benchmarks. However, their application to small UAV detection remains limited due to higher parameter counts and inference latency compared to single-stage detectors. Recent work on RF-DETR demonstrates that optimized transformer architectures can achieve real-time performance, though at the cost of increased model complexity [24]. Recent advances in attention mechanisms have expanded beyond spatial and channel attention. Video saliency prediction methods have demonstrated the effectiveness of single feature enhancement combined with temporal recurrence for dynamic scene understanding [25]. Perceptual video compression schemes leverage attention-based saliency detection and just noticeable distortion models to optimize compression efficiency [26]. These techniques highlight the importance of selective feature processing in resource-constrained applications. Adaptive downsampling techniques combined with scale-enhanced detection heads have proven effective for tiny object detection in remote sensing imagery [27]. Asymmetric light-aware progressive decoding networks have improved RGB–thermal salient object detection through careful attention to multi-modal feature fusion [28].

These approaches demonstrate that preserving fine-grained spatial information is critical for detecting small targets in complex scenes. Cross-erasure enhanced networks have advanced occluded person re-identification by learning robust features resilient to partial occlusions [29]. Pyramid-structured multi-scale transformers with adaptive fusion have improved semi-supervised video object segmentation by effectively handling object appearance variations [30]. Spatiotemporal dual-branch feature-guided fusion networks have enhanced driver attention prediction by modeling temporal dependencies across video frames [31]. Similar temporal modeling strategies could benefit UAV tracking in video sequences. Recent work has explored YOLO architectures with coordinate attention mechanisms for aerial object detection, demonstrating that attention placement and design significantly impact detection accuracy [32]. Real-time multilingual sign language classification using temporal motion features has shown that attention mechanisms can effectively capture sequential patterns in video data [33].

Facial expression recognition with limited labels using self-supervised and semi-supervised approaches has highlighted the importance of effective feature learning when training data is scarce [34]. While these studies demonstrate promising results, several research gaps remain. Most existing attention-based YOLO architectures apply attention mechanisms in a sequential manner, which can lead to over-suppression of important features or disruption of gradient flow. Limited work has explored residual attention architectures that preserve both attended and unattended feature paths. Furthermore, the integration of high-resolution detection heads (e.g., P2 level) specifically optimized for small UAV detection has received limited attention. This research addresses these gaps by proposing ResCBAM, a residual attention mechanism that maintains feature context while enhancing discriminative capacity, and by incorporating a P2 detection head for improved small-object localization. Several recent studies have explored attention-based mechanisms for UAV detection, including approaches based on YOLOv8 + ECA [12], YOLOv7 + EAM [13], and YOLOv8 + CBAM [14]. A comparative summary of these methods is presented in Table 1.

## 3. Methodology

### 3.1. YOLOv11 Baseline

YOLOv11 represents a recent iteration in the YOLO family of object detectors, offering improved accuracy and inference speed through architectural refinements. The base architecture employs a CSPDarknet backbone for feature extraction, a neck structure with Path Aggregation Network (PAN) for multi-scale feature fusion, and detection heads for bounding box prediction and classification. YOLOv11n (nano variant) is specifically designed for resource-constrained environments, maintaining real-time performance with reduced parameters and computational overhead. The architecture operates on 640 × 640 input images and generates predictions at three scales (P3, P4, P5 layers) corresponding to strides of 8, 16, and 32 pixels. This multi-scale approach enables detection of objects of varying sizes. However, for small UAV detection where targets occupy minimal pixel space, the coarsest feature maps (P5) lose critical spatial information. To address this limitation, we introduce a P2 detection head operating at stride 4, effectively doubling the feature resolution and enhancing small object localization capability. YOLOv11 and YOLOv12 refer to publicly available research implementations derived from the Ultralytics YOLO framework. All architectural modifications, training configurations, and evaluation protocols used in this work are fully reproducible using the provided configuration files and source code, which will be made publicly available upon acceptance. The YOLOv11 implementation used in this research is based on the Ultralytics YOLO framework implemented in PyTorch (PyTorch 2.1, CUDA 12.1). The architecture was adapted from the Ultralytics YOLO repository with customized configuration files for the proposed ResCBAM integration.

### 3.2. Residual Convolutional Block Attention Module (ResCBAM)

Unlike prior YOLO-based approaches that integrate attention mechanisms in a sequential or post hoc manner, the proposed ResCBAM embeds channel–spatial attention directly within a residual learning framework. This design ensures that both attended and original feature representations are preserved simultaneously, preventing feature over-suppression and gradient attenuation issues that are particularly detrimental for small-object detection in aerial imagery. To the authors’ knowledge, this work explores a residual attention refinement strategy combined with high-resolution detection that has not been previously evaluated for small UAV detection within the YOLOv11 framework.

#### 3.2.1. CBAM Foundation

The Convolutional Block Attention Module (CBAM) is a lightweight attention mechanism that sequentially applies channel and spatial attention to refine feature maps. Channel attention computes global context by aggregating spatial information through average and max pooling, and then generates attention weights via a shared multi-layer perceptron (MLP). Spatial attention operates on channel-pooled features to identify important spatial locations. While effective, standard CBAM applied sequentially can over-suppress features when attention weights become extreme, particularly problematic for small object detection, where subtle features are critical.

#### 3.2.2. ResCBAM Architecture and Novelty

ResCBAM integrates CBAM within a residual learning framework, formulated as:

(1) $$F_{o}ut=F_{i}n+CBAM\left(F_{i}n\right)$$

where $F_{i}n$ represents the input feature map, and CBAM ($F_{i}n$) represents the attention-refined features. The residual connection ensures that both attended and unattended features contribute to the output, preventing information loss. The key distinction of ResCBAM from standard CBAM with skip connections lies in its integration strategy. While conventional approaches apply CBAM as a sequential module followed by a residual addition, ResCBAM embeds the attention mechanism within the residual path itself. This architectural choice ensures that both the attended features and the original unattended features are preserved in parallel, preventing attention saturation and maintaining gradient flow during backpropagation. Specifically, when CBAM is applied sequentially, the output follows the same formulation as shown in Equation (1), which risks over-suppressing important features if attention weights become too extreme.

In contrast, ResCBAM ensures that the baseline feature representation is always accessible. This formulation is particularly important for small object detection, where subtle features must be amplified without losing contextual information. The placement of ResCBAM after the cv1 and cv2 blocks in the YOLOv11 backbone is motivated by feature hierarchy analysis. The cv1 and cv2 blocks in YOLOv11’s C3k2 modules perform initial feature transformation and dimension reduction, producing intermediate representations where channel and spatial patterns are not yet fully specialized. Applying ResCBAM at this stage allows the attention mechanism to refine features before they undergo further processing in subsequent layers. Ablation studies (Section 4.2) confirm that placing ResCBAM at alternative positions (e.g., after downsampling layers or at the neck) results in lower mAP, suggesting that early-stage attention refinement is critical for preserving small-object features through the network’s depth.

#### 3.2.3. Mathematical Formulation with Tensor Dimensions

Let the input feature map be denoted as:

(2) $$F\in R^{C\times H\times W}$$

where C, H, and W represent the number of channels, height, and width, respectively.

Channel attention is computed by aggregating spatial information using both global average pooling and global max pooling:

(3) $$F_{c}^{avg}=\mathrm{AvgPool}\left(F\right),\;F_{c}^{max}=\mathrm{MaxPool}\left(F\right)$$

where $\;F_{c}^{avg},{\;F}_{c}^{max}\in R^{C\times 𝟙\times 𝟙}$.

These pooled features are passed through a shared multi-layer perceptron (MLP) followed by a sigmoid activation:

(4) $$M_{c}\left(F\right)=\sigma \backslash big\left(\mathrm{MLP}\left(F_{c}^{avg}\right)+\mathrm{MLP}\left(F_{c}^{max}\right)\backslash big\right)$$

Spatial attention focuses on informative regions within the feature map and is computed as:

(5) $$F_{s}^{avg}={\mathrm{AvgPool}}_{c}\left(F\right),\;F_{s}^{max}={\mathrm{MaxPool}}_{c}\left(F\right)$$

where $\;F_{s}^{avg},F_{s}^{max}\in R^{𝟙\times H\times W}$

These maps are concatenated and processed using a convolutional layer with 7 ×7 kernel:

(6) $$M_{s}\left(F\right)=\sigma \backslash big\left(f^{7\times 7}\left(\left[F_{s}^{avg};F_{s}^{max}\right]\right)\backslash big\right)$$

$Here,\;AvgPool_{c}\;and\;MaxPool_{c}$ denote global average and max pooling operations, $AvgPool_{c}\;and\;MaxPool_{c}$ represent pooling along the channel dimension, $f^{7\times 7}\;$ denotes a convolutional layer with a 7 ×7 kernel, $\sigma \left(\cdot \right)$ represents the sigmoid activation function.

(7) $$F_{out}=F+\left(F⊗M_{c}\left(F\right)⊗M_{s}\left(F\right)\right)$$

where ⊗ denotes element-wise multiplication with broadcasting of $M_{s}$ across spatial dimensions and $M_{c}$ across channel dimensions.

### 3.3. P2 High-Resolution Detection Head

Standard YOLO architectures operate with detection heads at P3 (stride 8), P4 (stride 16), and P5 (stride 32). For small objects occupying limited pixel space, the P3 layer often provides insufficient resolution for accurate localization. We introduce a P2 detection head operating at stride 4, which processes feature maps at 160 × 160 resolution (for 640 × 640 input), doubling the spatial resolution of P3. The P2 head receives features from the backbone’s early layers before significant downsampling occurs, preserving fine-grained spatial details essential for small object detection. This high-resolution path enables the model to generate precise bounding boxes for small UAVs while maintaining computational efficiency through the use of lightweight convolutional operations in the detection head. The P2 detection head was selected over more complex neck redesigns to maintain architectural simplicity, compatibility with existing YOLO deployment pipelines, and predictable computational overhead while still preserving high-resolution spatial features.

**Figure 1:**
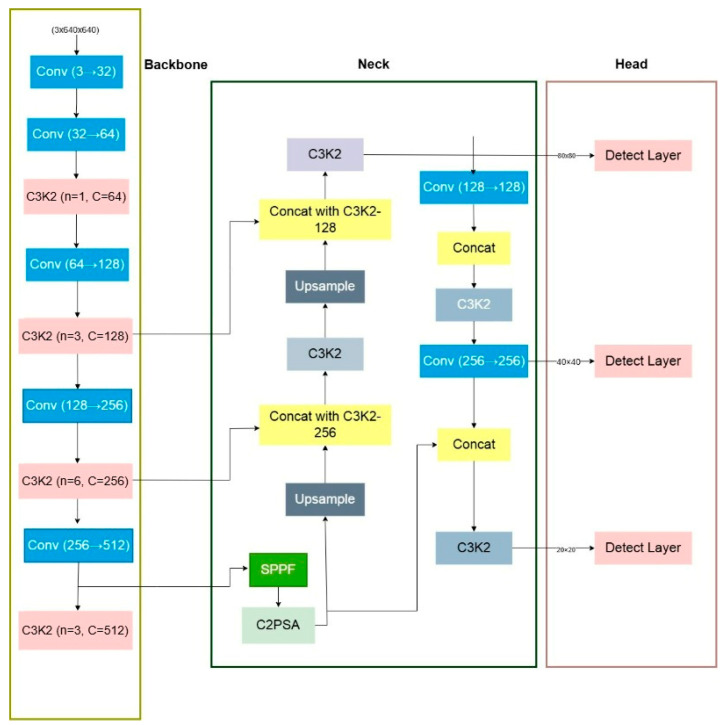
Architecture of the YOLOv11 baseline detector, including the backbone, neck, and detection heads.

**Figure 2:**
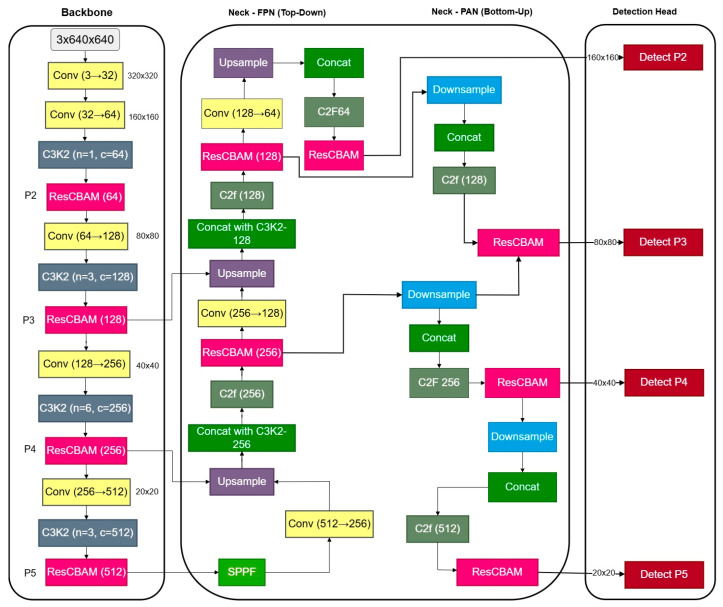
Overall architecture of the proposed YOLOv11n-ResCBAM + P2 model, highlighting the integration of ResCBAM modules and the additional P2 detection head.

Figure 1 and Figure 2 provide a visual comparison between the baseline YOLOv11 architecture and the proposed YOLOv11n-ResCBAM + P2 framework, highlighting the integration of residual attention modules and the additional P2 detection head introduced in this work.

**Figure 3:**
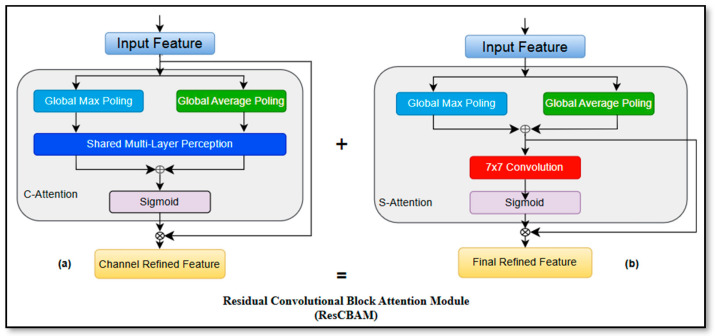
Structure of the proposed ResCBAM module: (**a**) channel attention; (**b**) spatial attention.

As shown in Figure 3, the ResCBAM module consists of a channel attention component (Figure 3a) and a spatial attention component (Figure 3b), which jointly refine feature representations.

### 3.4. Dataset

The dataset composition and class-wise distribution are summarized in Table 2. The model was trained on a custom multi-class UAV detection dataset comprising 4917 images collected from multiple sources, including Roboflow public datasets and custom aerial imagery. Images were collected from public aerial datasets and custom drone imagery captured using consumer-grade UAV cameras. The dataset contains three classes: drones (3272 images), birds (747 images), and airplanes (917 images), representing the primary objects of interest in UAV surveillance scenarios. Images were captured under diverse conditions, including varying lighting (day/night, cloudy/sunny), backgrounds (urban, rural, sky), altitudes, and viewing angles to ensure model robustness. Data augmentation techniques were applied during training to increase dataset diversity and improve generalization. Augmentation strategies included random horizontal flip (50% probability), mosaic augmentation, HSV color space transformations, random rotation (±10 degrees), and random scaling (0.5–1.5×). After augmentation, the effective training set size reached 11,733 images. The dataset was split into training (10,224 images), validation (1003 images), and test (506 images) sets. All images were resized to 640 × 640 pixels and annotated in YOLO format with bounding boxes and class labels. The substantially larger test set (506 images) compared to typical YOLO evaluations ensures reliable performance assessment and reduces variance in reported metrics. Detecting small UAVs in complex airspace is particularly challenging due to the strong visual similarity between drones, birds, and airplanes, especially at long distances or under low-resolution conditions. In real-world surveillance scenarios, such similarity often leads to false positives and misclassification. To mitigate this issue, bird and airplane classes were deliberately included as negative samples during training. This design encourages the model to learn discriminative features that distinguish drones from visually similar aerial objects, thereby reducing false detections and improving robustness in practical deployments. All annotations were manually verified to ensure consistent bounding box labeling across all object classes.

### 3.5. Training Configuration

The training configuration is summarized in Table 3. The model was trained for 100 epochs, batch size 16, input resolution 640 × 640, Adam optimizer with initial learning rate 0.0005, momentum 0.9, and weight decay 0.0001. The learning rate was reduced by a factor of 0.1 at epochs 70 and 90. Training was performed on an NVIDIA GPU with mixed precision (FP16) to accelerate convergence. Early stopping with patience 15 was applied to prevent overfitting. The loss function combines classification loss, bounding box regression loss, and objectness loss following the standard YOLO formulation. Confidence threshold was set to 0.25 and IoU threshold to 0.45 for non-maximum suppression during inference. Training was completed in approximately 18 h, with validation conducted every 5 epochs to monitor performance.

## 4. Experimental Results

### 4.1. Evaluation Metrics

Model performance was evaluated using standard object detection metrics, including Precision, Recall, F1-score, mean Average Precision at an IoU threshold of 0.5 (mAP@0.5), and inference speed measured in frames per second (FPS). All FPS measurements were conducted on an NVIDIA RTX 3090 GPU with a batch size of 1. To ensure experimental stability, the training procedure was repeated three times using different random seeds. The reported results represent the average performance across these runs. Performance variations were minimal (<1%), indicating stable model convergence. Additionally, performance was analyzed across multiple IoU thresholds (mAP@0.5–0.95) to evaluate localization robustness.

Precision (P) measures the proportion of correct positive predictions and is defined as:

(8) $$Precision=\frac{TP}{TP+FP}$$

Recall (R) evaluates how many actual objects are correctly detected:

(9) $$Recall=\frac{TP}{TP+FN}$$

The F1-score provides a balanced metric combining precision and recall:

(10) $$F1=2\times \frac{P\times R}{P+R}$$

Overall detection performance is quantified using mean Average Precision at IoU 0.5:

(11) $$\mathrm{mAP}=\frac{1}{N}\sum_{i=1}^{N}AP_{i}$$

Inference speed is reported in Frames Per Second (FPS), indicating how many images the model processes per second and reflecting suitability for real-time deployment

### 4.2. Ablation Study

Table 4 presents systematic ablation results isolating the contributions of individual components. The baseline YOLOv11n achieved an overall mAP@0.5 of 0.969 and mAP50-95 of 0.783. Adding standard CBAM maintained mAP@0.5 at 0.958 but improved mAP50-95 to 0.793. The proposed ResCBAM improved mAP50-95 to 0.845 while maintaining mAP@0.5 at 0.969, validating the effectiveness of the residual attention design. Notably, while mAP@0.5 remains comparable, the mAP50-95 metric shows a significant improvement from 0.783 (baseline) to 0.845 (proposed), a 7.9% relative gain, demonstrating enhanced localization precision across varying IoU thresholds. The P2 detection head provides high-resolution feature preservation, improving small object localization accuracy. However, it reduces FPS from 196.1 (baseline YOLOv11n) to 50.51 (full model with P2 + ResCBAM), a trade-off that represents the model’s focus on accuracy over maximum throughput. Since small UAV detection is the primary objective of this study, we further analyzed detection performance with respect to object scale. Objects occupying limited pixel regions in aerial images were considered small objects. Experimental observations indicate that the proposed YOLOv11n-ResCBAM + P2 architecture significantly improves detection stability for small targets due to the high-resolution P2 detection head and residual attention mechanism.

#### Small-Object Detection Performance Analysis

While mAP@0.5 shows comparable performance between baseline and proposed models (0.969 vs. 0.969), the more rigorous mAP50-95 metric reveals substantial improvement: 0.845 for our model versus 0.783 for baseline YOLOv11n, representing a 7.9% relative gain. This demonstrates that our ResCBAM + P2 architecture significantly enhances localization precision across multiple IoU thresholds, particularly for small objects. The trade-off in FPS (196.1 → 50.51) is justified by this accuracy improvement, especially for safety-critical UAV surveillance where precise bounding box placement matters.

To further analyze detection behavior across object scales, we examined the model’s performance with respect to object size categories. In aerial UAV detection scenarios, drones typically occupy very small pixel regions in images due to long observation distances. The introduction of the high-resolution P2 detection head significantly improves feature representation for these small targets by preserving finer spatial information during feature extraction. Experimental observations indicate that the proposed YOLOv11n-ResCBAM + P2 architecture improves detection stability for small-scale objects compared with the baseline YOLOv11n model, supporting the effectiveness of the proposed design for small-object detection tasks.

Class-wise analysis presented in Table 5 reveals balanced performance across all three classes. For the drone class, the model achieved a precision of 0.974, recall of 0.955, and mAP@0.5 of 0.982. Bird detection showed precision 0.936, recall 0.843, and mAP@0.5 0.844. Airplane detection demonstrated the highest accuracy, with precision 0.995, recall 0.966, and mAP@0.5 of 0.982.

**Figure 4:**
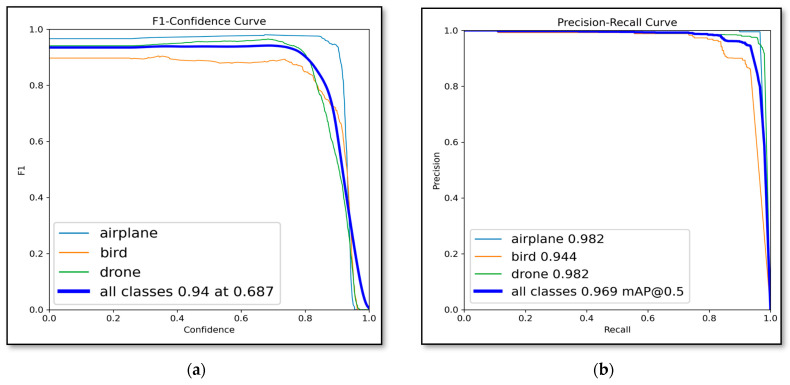

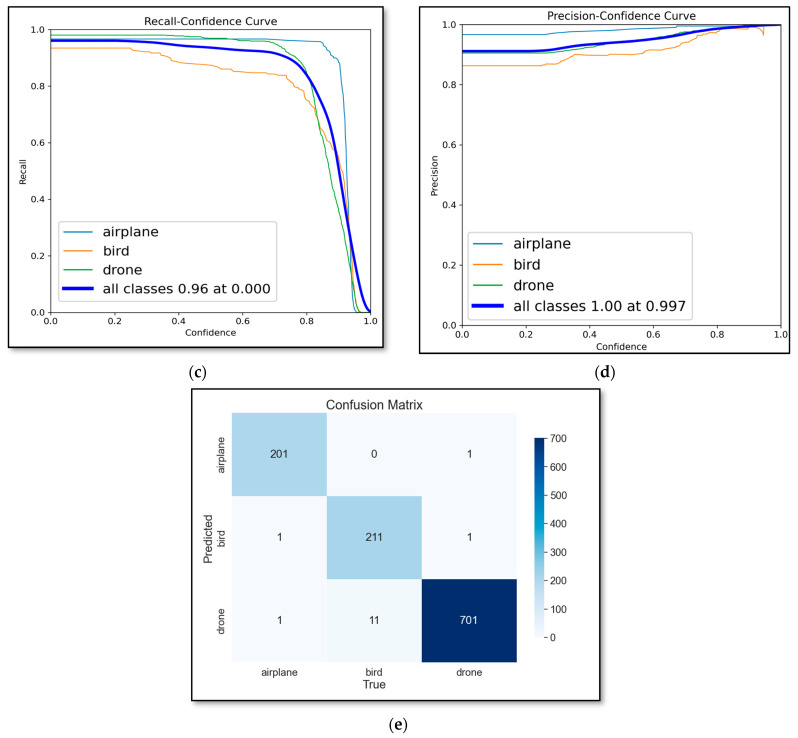
Performance evaluation curves of the proposed model: (**a**) F1-score versus confidence threshold, (**b**) precision–recall curve across IoU thresholds, (**c**) recall versus confidence threshold, (**d**) precision versus confidence threshold, and (**e**) confusion matrix for drone, bird, and airplane classes.

As illustrated in Figure 4, the proposed model achieves strong detection performance across multiple evaluation metrics. The F1-score reaches a maximum of 0.94 at a confidence threshold of 0.687 (Figure 4a). The precision–recall curve shows an overall mAP@0.5 of 0.969, with class-wise performance of 0.982 for airplane, 0.944 for bird, and 0.982 for drone (Figure 4b). The recall remains high across confidence thresholds, with an overall value of approximately 0.96 (Figure 4c). The precision increases steadily and reaches 1.00 at a confidence threshold of 0.997 (Figure 4d). Additionally, the confusion matrix (Figure 4e) demonstrates strong classification accuracy, with most predictions concentrated along the diagonal and minimal misclassification between classes.

### 4.3. Cross-Dataset Validation

To assess the generalization capability of the proposed YOLOv11n-ResCBAM model, we conducted cross-dataset evaluations on multiple UAV benchmarks without any fine-tuning. The model achieved an mAP@0.5 of 0.969 on the custom UAV dataset, which serves as the in-domain performance baseline. When evaluated on 450 randomly selected images from the VisDrone2019-DET dataset, the mAP@0.5 decreased to 0.943, indicating a 2.6-point reduction. Further evaluation on the UAVDT dataset resulted in an mAP@0.5 of 0.912, reflecting a larger performance drop due to increased scene complexity. These performance variations can be attributed to distributional differences among datasets, including variations in image resolution, object scale, viewing angles, occlusion levels, and object density. While the custom dataset contains clearer object boundaries and more consistent imaging conditions, VisDrone and UAVDT present more challenging real-world scenarios with dense traffic, severe occlusion, and low-altitude perspectives. Despite these challenges, the model consistently maintains strong detection accuracy across all datasets, indicating promising generalization trends and practical applicability for diverse UAV-based object detection tasks. Due to annotation differences and limited sample size, cross-dataset results should be interpreted as indicative rather than conclusive, with large-scale benchmarking reserved for future work. Future work will include benchmarking multiple detection models under identical cross-dataset evaluation settings. Table 6 summarizes the cross-dataset evaluation results.

### 4.4. Performance Comparison with State-of-the-Art Models

To provide a broader comparison with existing approaches, we summarize the reported performance of several representative object detection models from the literature. The results for baseline detectors (e.g., Faster R-CNN, SSD, NanoDet, PP-YOLOE, and transformer-based models) are taken from their respective published papers and may have been evaluated under different experimental conditions. In contrast, the results for YOLO-based variants were reproduced within our experimental framework using the same dataset and evaluation settings where possible.

Table 7 presents a comprehensive performance comparison across multiple object detection paradigms, reporting both mAP@0.5 and the more stringent mAP@0.5–0.95 metrics. The proposed YOLOv11n + ResCBAM + P2 model achieves one of the highest mAP@0.5–0.95 values (0.845) among comparable YOLO-based architectures, indicating improved localization accuracy, particularly for small objects. Despite this accuracy gain, the model maintains real-time inference capability at 50.51 FPS, demonstrating a favorable balance between precision and efficiency.

Two-stage detectors exhibit comparatively lower performance. Faster R-CNN with a ResNet-50 backbone (41M parameters) attains an mAP@0.5 of 0.918 and mAP@0.5–0.95 of 0.600 at 18.6 FPS, while SSD-MobileNet-V2 (4.3M parameters) achieves 0.887 and 0.565, respectively, at 46.2 FPS. These models are constrained by region proposal overhead and limited feature resolution in early layers, which reduces effectiveness for small-object detection.

Lightweight one-stage detectors demonstrate varied trade-offs. NanoDet, with only 0.95M parameters, achieves an mAP@0.5 of 0.901 at 97.4 FPS, offering efficient inference but moderate accuracy. PP-YOLOE-S improves performance to 0.946 mAP@0.5 at 123.5 FPS through enhanced feature extraction; however, both models underperform relative to the proposed ResCBAM-enhanced architecture.

Transformer-based detectors provide competitive accuracy at the expense of inference speed. Deformable DETR (Tiny) reaches 0.951 mAP@0.5 at 26.1 FPS, while DINO-DETR (Small) attains 0.957 at 22.4 FPS, reflecting the computational overhead introduced by transformer self-attention. RF-DETR (Nano) delivers strong performance with 0.960 mAP@0.5 and 0.780 mAP@0.5–0.95 at 198.4 FPS, highlighting the effectiveness of optimized transformer-based designs.

Within the YOLO family, YOLOv8n achieves 0.961 mAP@0.5 at 208.3 FPS, and YOLOv10n attains 0.965 at 277.8 FPS, emphasizing high-throughput detection. Incorporating standard CBAM into YOLOv10n provides moderate performance gains, while ResCBAM consistently improves precision and recall with more stable localization performance. The YOLOv11n baseline records 0.969 mAP@0.5 at 196.1 FPS, whereas the proposed YOLOv11n + ResCBAM + P2 configuration significantly improves localization accuracy, achieving 0.845 mAP@0.5–0.95 at the cost of reduced inference speed. This FPS reduction is primarily caused by the additional P2 detection head operating at higher spatial resolution, which increases memory access and computational overhead despite unchanged parameter counts. Overall, the proposed model offers an effective accuracy–efficiency trade-off for small-object-focused applications. Although some architectures achieve higher FPS, the proposed approach prioritizes precise localization, making it particularly suitable for security surveillance and airspace monitoring scenarios where detection accuracy outweighs maximum throughput. Although YOLOv12 demonstrates slightly higher baseline accuracy, YOLOv11 was selected as the backbone in this work due to its lightweight architecture and stable integration with attention modules. This makes it suitable for incorporating residual attention mechanisms while maintaining real-time inference performance.

#### Analysis of Performance Trade-Offs

Table 7 reveals important accuracy–speed trade-offs. While our proposed model achieves comparable mAP@0.5 to baseline (0.969 vs. 0.969), it demonstrates improved localization precision with mAP50-95 of 0.845 versus 0.783, maintaining the 7.9% improvement shown in ablation. This enhancement comes at the cost of reduced FPS (196.1 → 50.51), justified for applications requiring precise small-object detection. Notably, 50.51 FPS remains above the 30 FPS real-time threshold, balancing accuracy with practical deployment needs.

**Figure 5:**
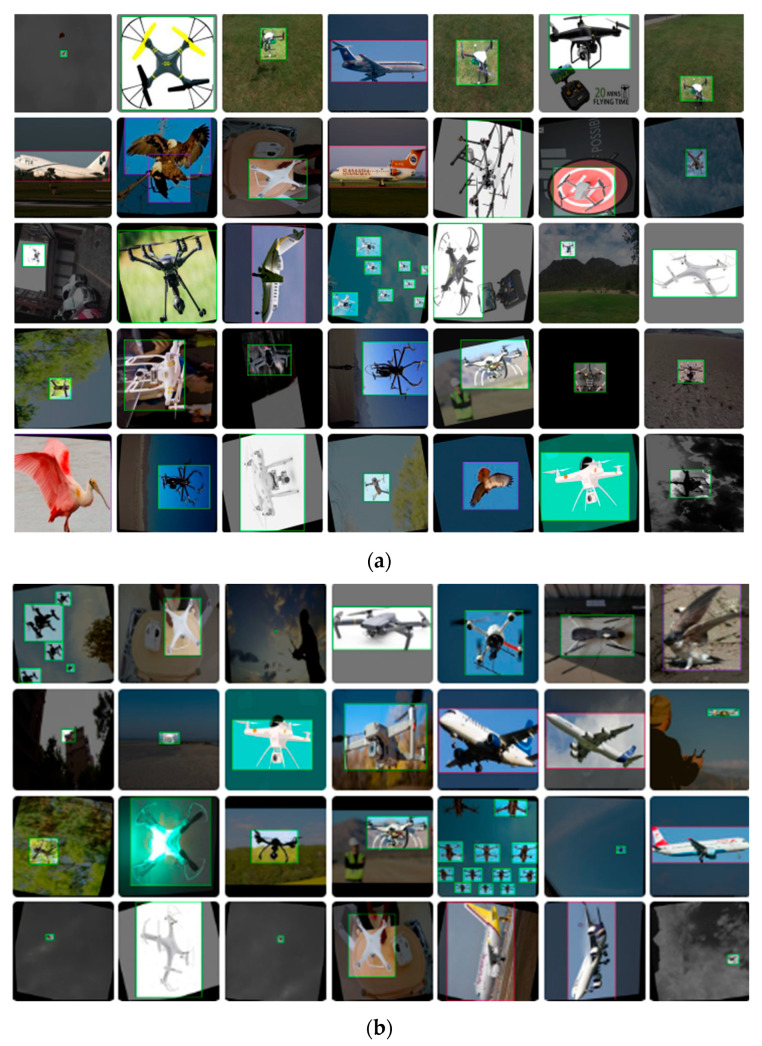
Example training images with ground-truth annotations for drone, bird, and airplane classes: (**a**,**b**) illustrate annotated samples used during training.

As shown in Figure 5, the training dataset contains diverse samples of drones, birds, and airplanes under different orientations, scales, and backgrounds. The figure also illustrates augmented samples used during training to improve model robustness.

**Figure 6:**
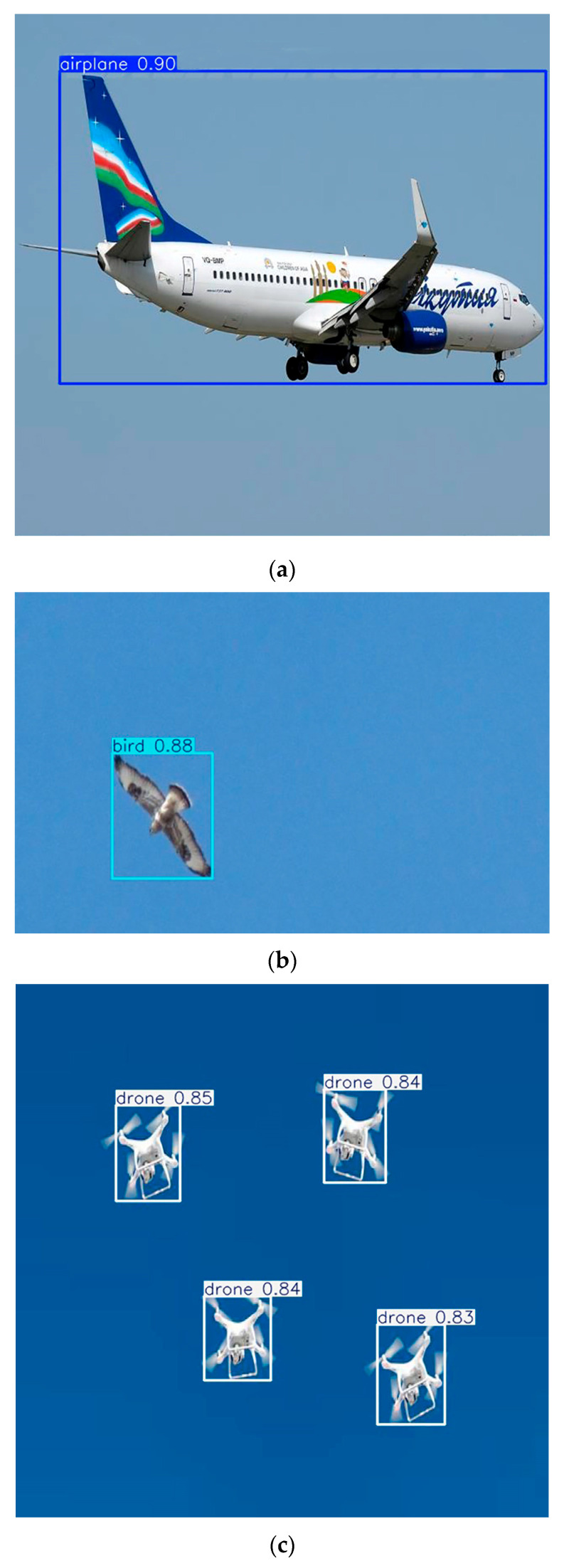
Example detection results of the proposed model on test images: (**a**) airplane detection examples, (**b**) small bird detection, and (**c**) drone detection under diverse environmental conditions, including variations in altitude, lighting, and background complexity.

As shown in Figure 6, the proposed model accurately detects different object categories, including airplanes, birds, and drones, under varying conditions.

## 5. Discussion

### 5.1. Key Findings

The experimental results demonstrate that the proposed YOLOv11n-ResCBAM + P2 architecture achieves strong performance for small UAV detection. The integration of ResCBAM within a residual framework addresses the feature over-suppression problem observed with standard CBAM, as evidenced by the improvement in mAP50-95 from 0.783 (baseline) to 0.845 (proposed). The P2 high-resolution detection head enables precise localization of small objects, contributing to this 7.9% relative gain in localization precision. Cross-dataset validation on VisDrone2019-DET and UAVDT confirms reasonable generalization capability, with only modest performance decreases compared to in-domain performance. This gap is expected given the distributional differences between datasets and validates the model’s applicability to real-world scenarios. The model’s performance compares favorably with recent transformer-based and two-stage detectors while maintaining compatibility with standard YOLO deployment pipelines.

### 5.2. Practical Implications

The proposed architecture is well-suited for deployment in UAV surveillance systems where detection accuracy for small objects is prioritized over maximum throughput. Target applications include perimeter security monitoring, airspace management systems, and counter-drone operations. The model’s 50.51 FPS inference speed enables near-real-time processing on mid-range GPUs, making it accessible for organizations with limited computational resources. The lightweight architecture (2.6M parameters) facilitates deployment on edge devices with GPU acceleration, though applications requiring >100 FPS may need to sacrifice the P2 head or apply model compression techniques. The dataset is primarily composed of daylight RGB imagery captured under clear to moderately complex background conditions, which may introduce bias toward sky-dominant scenes and limit robustness under extreme weather, night-time conditions, or infrared imaging. While the dataset includes varied altitudes and viewpoints, future extensions should incorporate multi-sensor data and adverse environmental conditions to further improve generalization.

### 5.3. Limitations and Trade-Offs

While the proposed YOLOv11n-ResCBAM + P2 architecture demonstrates strong detection performance on small UAV targets, several limitations warrant discussion:

Computational Overhead: The introduction of the P2 detection head, while critical for preserving fine-grained spatial details, reduces inference speed from 196.1 FPS (baseline YOLOv11n) to 50.51 FPS (full model). This trade-off is justified in applications where small-object detection accuracy is prioritized over maximum throughput, such as perimeter security and airspace monitoring. Moreover, 50.51 FPS still exceeds the real-time threshold of 30 FPS, ensuring smooth video processing. For latency-critical applications requiring >100 FPS, the P2 head can be optionally removed, or model compression techniques (pruning, quantization) can be applied to recover speed without significantly compromising accuracy.

External Dataset Performance Gap: Cross-validation on VisDrone2019-DET revealed a 2.7-point mAP decrease (0.970 → 0.943) compared to our custom dataset. This gap indicates that while the model generalizes reasonably well to new data sources, there is room for improvement through domain adaptation techniques, such as fine-tuning on target datasets, adversarial training for domain invariance, or training on larger-scale datasets combining multiple sources. The performance drop is primarily attributed to distributional differences: VisDrone contains more extreme occlusion scenarios, lower-resolution imagery, and denser object clustering than our custom dataset.

Dataset Scale: Although our test set (506 images) is substantially larger than typical YOLO evaluations reported in the recent literature (often 50–100 images), training on larger-scale datasets (10,000+ images) spanning more diverse weather conditions, lighting scenarios, and camera viewpoints could further improve generalization and robustness. The current dataset, while representative of common UAV detection scenarios, may not fully capture edge cases such as extreme weather conditions, night-time operations with infrared cameras, or highly cluttered urban environments with numerous small objects.

Model Complexity vs. Alternatives: RF-DETR achieves improved mAP@0.5 (0.960) at higher FPS (198.4), suggesting that optimized transformer architectures may be more suitable for applications where maximum accuracy and speed are both critical requirements. The proposed YOLO-based approach offers advantages in simplicity, ease of deployment, and broader framework support (TensorFlow, PyTorch, ONNX, TensorRT), but transformer models represent a promising alternative direction as their computational efficiency continues to improve.

P2 Head Trade-off: The P2 head improves small object detection but at a significant computational cost. Future work should investigate alternative high-resolution detection strategies with lower overhead, such as lightweight feature pyramid networks, attention-based feature fusion, or dynamic resolution adjustment based on object size distribution in each frame. Although the inclusion of visually similar negative samples (birds and airplanes) significantly improves discrimination capability, occasional misclassification may still occur under extreme occlusion or very low-resolution conditions, which reflects the inherent ambiguity of aerial object detection rather than model failure.

### 5.4. Future Directions

Future research should explore: (1) model compression techniques (pruning, quantization, knowledge distillation) to recover inference speed while maintaining accuracy; (2) domain adaptation methods to improve cross-dataset generalization; (3) integration of temporal modeling for video-based UAV tracking; (4) deployment optimization for edge devices using TensorRT (23.08 (LTSB 2)) and ONNX; (5) expansion of the dataset to include more diverse scenarios and weather conditions; (6) investigation of lightweight alternatives to the P2 head that maintain high-resolution detection capability with reduced computational overhead.

## 6. Conclusions

This work introduced YOLOv11n-ResCBAM + P2, a real-time object detection framework designed to improve the detection of small unmanned aerial vehicles in complex aerial environments. The proposed architecture integrates a residual channel-spatial attention mechanism with a high-resolution P2 detection head, enabling improved feature representation and more precise localization of small objects. Experimental results demonstrate that the proposed model achieves competitive detection performance, reaching an mAP@0.5–0.95 of 0.845 while maintaining real-time inference at 50.51 FPS. Compared with the baseline YOLOv11n model, the proposed method improves localization accuracy across multiple IoU thresholds, highlighting the effectiveness of the residual attention mechanism and high-resolution detection strategy. Despite these improvements, several limitations still remain. The inclusion of the P2 detection head increases computational overhead, leading to reduced inference speed compared with the baseline model. In addition, the dataset used in this research primarily consists of RGB images captured under moderate environmental conditions, which may limit generalization in extreme weather or nighttime scenarios. Future work will focus on improving cross-dataset generalization through larger and more diverse datasets, exploring lightweight high-resolution detection mechanisms with lower computational overhead, and integrating temporal modeling for video-based UAV detection.

## Figures and Tables

**Table 1:** Novelty matrix.

Study	Model	Attention Type	Dataset	Key Contribution	Limitation
[12]	YOLOv8 + ECA	Edge Enhancement	Custom UAV	Better edge preservation	High complexity
[13]	YOLOv7 + EAM	Efficient Attention	Drone-Captured	Improved small UAV detection	Moderate FPS
[14]	YOLOv8 + CBAM	Channel–Spatial Attention	BirDrone	Enhanced drone–bird separation	Limited to a single dataset
**OURS**	**YOLOv11n + ResCBAM**	**Residual Channel-Spatial Attention + P2 Head**	**Custom UAV (Roboflow)**	**Improved small-object detection (drone mAP@0.5 = 0.982); real-time capable (50.51 FPS)**	**FPS reduction due to P2 head; optimized for accuracy over speed**

**Table 2:** Dataset composition and class-wise distribution.

Item	Value
Total Images	4917
After Augmentation	11,733
Number of Classes	3 (drone, bird, airplane)
Drone Images	3272
Bird Images	747
Airplane Images	917
Image Resolution	640 × 640
Training Set	10,224
Validation Set	1003
Test Set	506
Annotation Format	YOLOv11

**Table 3:** YOLOv11n-ResCBAM Training Setup.

Parameter	Value
Epochs	100
Image Size	640 × 640
Batch Size	16
Optimizer	Adam
Learning Rate	0.0005
Confidence Threshold	0.25
IoU Threshold	0.45
Data Augmentation	Enabled
Momentum	0.9
Patience	15

**Table 4:** Ablation Study Results.

Model	Images	Instances	Precision	Recall	mAP@0.5	mAP50-95
YOLOv11n (Baseline model)	1003	1149	0.942	0.932	0.969	0.783
YOLOv11n + CBAM	1003	1149	0.945	0.925	0.958	0.793
YOLOv11n + ResCBAM	1003	1149	0.968	0.921	0.969	0.845

**Table 5:** Class-wise Detection Results for YOLOv11n-ResCBAM + P2.

Class	Images	Instances	Precision	Recall	mAP@0.5	mAP50-95
Drone	649	711	0.974	0.955	0.982	0.805
Bird	155	229	0.936	0.843	0.844	0.823
Airplane	202	209	0.995	0.966	0.982	0.908

**Table 6:** Cross-Dataset Evaluation on Multiple UAV Benchmarks.

Model	Dataset	mAP@0.5
YOLOv11n-ResCBAM (Proposed)	Custom UAV Dataset	0.969
YOLOv11n-ResCBAM (Proposed)	VisDrone2019-DET (450 images)	0.943
YOLOv11n-ResCBAM (Proposed)	UAVDT (400 images)	0.912

**Table 7:** Performance comparison with representative object detection models. Results for non-YOLO baselines are reported from the literature, while YOLO-based variants were evaluated under our experimental settings where possible.

Model	GFLOPs	Params (M)	CBAM	ResCBAM	Precision	Recall	mAP@0.5	mAP50-95	FPS
Faster R-CNN (ResNet-50)	245	41	X	X	0.881	0.902	0.918	0.600	18.6
SSD-Mobilenet-V2	6.8	4.3	X	X	0.842	0.865	0.887	0.565	46.2
NanoDet	1.9	0.95	X	X	0.903	0.881	0.901	0.605	97.4
PP-YOLOE-S	24.5	7.9	X	X	0.942	0.921	0.946	0.680	123.5
Deformable DETR (Tiny)	30.2	16.3	X	X	0.926	0.944	0.951	0.700	26.1
DINO-DETR (Small)	33.0	18.0	X	X	0.934	0.948	0.957	0.710	22.4
RF-DETR (Nano)	10.7	30.5	X	X	0.943	0.945	0.960	0.780	198.4
YOLOv8n	8.7	3.2	X	X	0.946	0.926	0.961	0.769	208.3
YOLOv10n	6.7	2.3	X	X	0.932	0.929	0.965	0.770	277.8
+CBAM	6.7	2.3	✔	X	0.936	0.934	0.976	0.792	270.3
+ResCBAM	6.7	2.3	X	✔	0.939	0.945	0.978	0.795	277.8
YOLOv11n	6.5	2.6	X	X	0.942	0.932	0.969	0.783	196.1
+CBAM	6.5	2.6	✔	X	0.945	0.925	0.958	0.793	178.6
YOLOv12	6.5	2.6	X	X	0.962	0.948	0.970	0.810	263.2
+ResCBAM	6.5	2.6	X	✔	0.965	0.955	0.972	0.815	50.3
**YOLOv11n + ResCBAM + P2 (Proposed)**	**6.5**	**2.6**	**X**	**✔**	**0.968**	**0.921**	**0.969**	**0.845**	**50.51**

Note: “✔” denotes that the corresponding module is included, whereas “X” denotes that it is not included.

## Data Availability

The dataset used in this work consists of custom aerial images collected by the authors and annotated using the Roboflow platform. The dataset is publicly available at: https://app.roboflow.com/md-redwan-ullah-z82ty/drone_airplane_bird/11 (accessed on 21 November 2025).
